# Overview of global publications on machine learning in diabetic retinopathy from 2011 to 2021: Bibliometric analysis

**DOI:** 10.3389/fendo.2022.1032144

**Published:** 2022-12-15

**Authors:** An Shao, Kai Jin, Yunxiang Li, Lixia Lou, Wuyuan Zhou, Juan Ye

**Affiliations:** ^1^ Department of Ophthalmology, the Second Affiliated Hospital of Zhejiang University, College of Medicine, Hangzhou, China; ^2^ College of Computer Science and Technology, Hangzhou Dianzi University, Hangzhou, China; ^3^ Zhejiang Academy of Science and Technology Information, Hangzhou, China

**Keywords:** machine learning, diabetic retinopathy, global publication trend, topic analysis, bibliometric analysis

## Abstract

**Purpose:**

To comprehensively analyze and discuss the publications on machine learning (ML) in diabetic retinopathy (DR) following a bibliometric approach.

**Methods:**

The global publications on ML in DR from 2011 to 2021 were retrieved from the Web of Science Core Collection (WoSCC) database. We analyzed the publication and citation trend over time and identified highly-cited articles, prolific countries, institutions, journals and the most relevant research domains. VOSviewer and Wordcloud are used to visualize the mainstream research topics and evolution of subtopics in the form of co-occurrence maps of keywords.

**Results:**

By analyzing a total of 1147 relevant publications, this study found a rapid increase in the number of annual publications, with an average growth rate of 42.68%. India and China were the most productive countries. *IEEE Access* was the most productive journal in this field. In addition, some notable common points were found in the highly-cited articles. The keywords analysis showed that “diabetic retinopathy”, “classification”, and “fundus images” were the most frequent keywords for the entire period, as automatic diagnosis of DR was always the mainstream topic in the relevant field. The evolution of keywords highlighted some breakthroughs, including “deep learning” and “optical coherence tomography”, indicating the advance in technologies and changes in the research attention.

**Conclusions:**

As new research topics have emerged and evolved, studies are becoming increasingly diverse and extensive. Multiple modalities of medical data, new ML techniques and constantly optimized algorithms are the future trends in this multidisciplinary field.

## Introduction

Diabetic retinopathy (DR), as one of the characterized microvascular complications of diabetes mellitus, has already become the leading cause of vision loss in the worldwide working-age population (1). Most patients with early-stage DR appear normal without any visual disruptions, however, the potential pathological changes, such as microvascular damage and neurodegeneration, are progressing (2, 3). Severe DR can cause visual impairment and finally lead to irreversible blindness, seriously affecting the quality of life. To prevent or manage DR, screening, early detection and intervention are crucial (4). In clinical practice, fundus examinations are recommended during the process of screening, diagnosis and follow-up of DR. The mainstream examinations include digital retinal photography, optical coherence tomography (invasive technologies such as fundus fluorescein photography are less common), etc. (1, 5, 6). Ophthalmologists can diagnose DR based on the typical lesions (e.g., exudates, microaneurysms) that appeared in the digital images (7). In addition to forming the basis of clinical diagnosis, the massive medical data from examinations has significant value for academic research.

With the development of artificial intelligence (AI) technologies, machine learning (ML), as an advanced field of AI, has gradually intertwined with various aspects of modern medicine (8). Machine learning focuses on enabling computers to automatically learn from the data of different modalities without being explicitly programmed (9). ML is a general name including many technological terms, such as deep learning (DL), supervised learning or neural networks. The implementation of ML in medicine is usually related to disease detection, survival prediction and risk evaluation, and so on (10–12). When compared to other medical specialties, ophthalmology features a wide application of imaging techniques with abundant data resources and an urgent need for computer-aided diagnosis due to the shortage of ophthalmologists (13). This leads to the emergence and rapid development of ML in ophthalmology. DR is one of the widely researched diseases in this field because of its increasing prevalence and the high risk of blindness in severe cases. Automatic DR grading/identification, automatic DR lesion detection and other related achievements have been reported in various conferences or journal articles (14, 15). Moreover, a number of review articles discuss the overall development of ML techniques in DR (4, 16). Thus far, however, no bibliometric analysis has been conducted on this topic.

The bibliometric analysis uses mathematical and statistical methodologies to obtain quantifiable and objective data from intangible features of the literature (17, 18). It has been applied in numerous topics and disciplines. To our knowledge, this is the bibliometric study focused on the literature related to ML in DR. To search for as much relevant literature as possible, we prepared a keyword list based on related books and articles. However, the search based on these keywords leads to the retrieval of documents with diversified purposes, study design and topics, or some irrelevant records. Thereby we generated the inclusion criteria and manually screened all the retrieved documents to confirm that included articles focused on ML in DR. Moreover, as the topics of included documents are relatively diverse and impractical to summarize one by one, we utilized VOSviewer and Wordcloud to visualize these topics in the form of co-occurrence maps of keywords. In addition, we also interpret our results based on the overall progress of ML techniques and the status of DR during 2011-2021 to make our analysis reasonable.

This paper has three objectives: first, to summarize the publication trend and identify the outstanding achievements; second, to reveal the contributions of countries/institutions/journals and visualize the collaboration networks; third, to uncover the mainstream topic and study the evolution of subtopics in this area.

## Methods

### Search strategy

**Figure 1:**
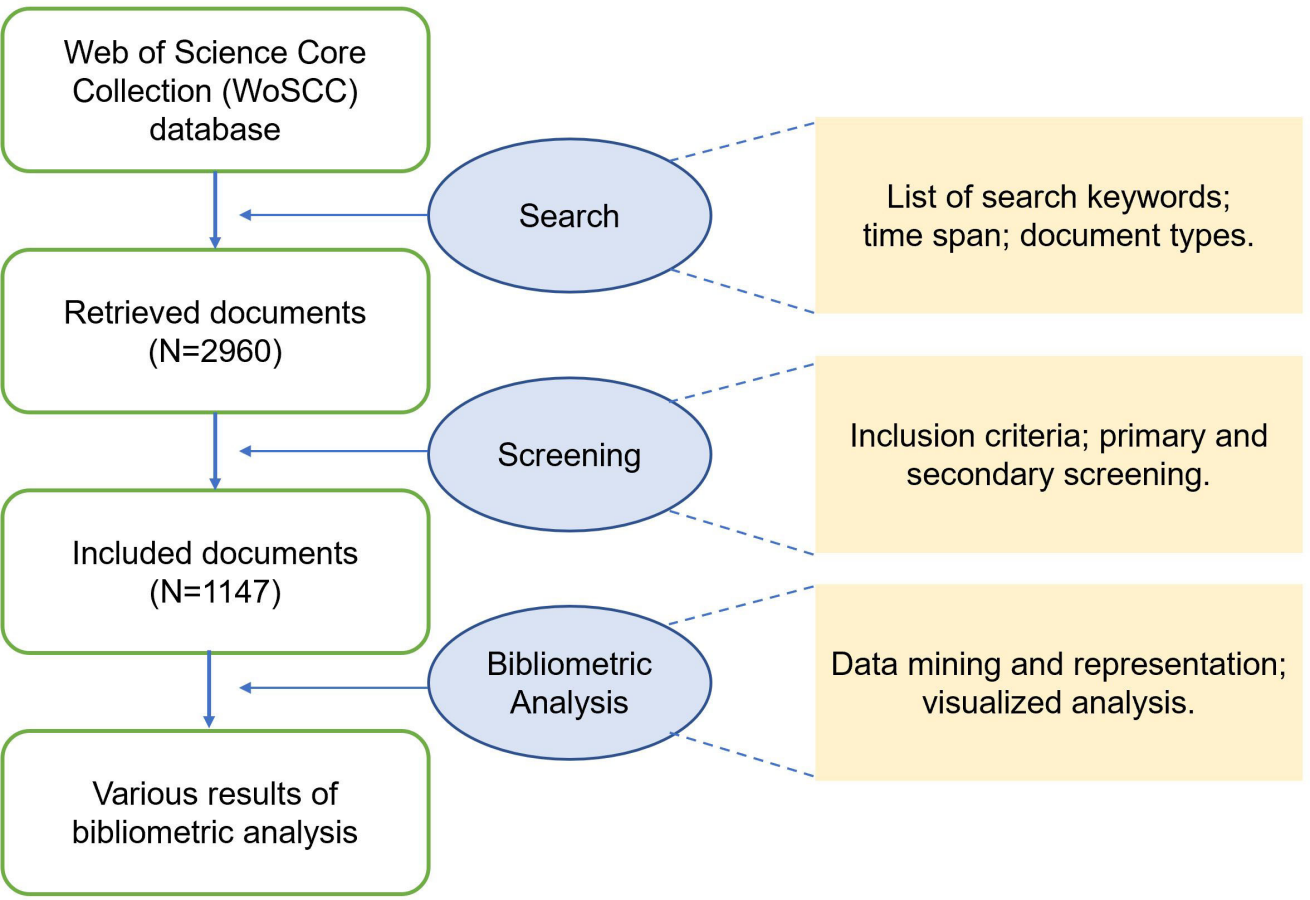
Flowchart of search, screening and bibliometric analysis.

All of the reference data used in this study were collected from the Web of Science Core Collection (WoSCC), which incorporates articles in over 20,000 high-quality peer-reviewed scholarly journals published worldwide in addition to a large number of proceedings papers (a single set of 28 criteria was made to evaluate journals). To search for the relevant data, a set of DR-related keywords and a set of ML-related keywords were prepared based on relevant literature and books (1, 19). Specific keywords were shown in **Table 1**. We searched for documents containing at least one DR-related keyword and one ML-related keyword in the “topic” of records (including title, abstract, author and keywords), for example, the documents that include both “diabetic retinopathy” and “machine learning”. As many state-of-the-art achievements in computer science involving machine learning technologies would publish in conference proceedings besides journal articles, the scope of document types included journal articles, proceedings papers and reviews. The timespan was from 2011 to 2021. The last search was conducted on September 24, 2021. A total of 2960 retrieved documents from the WoSCC were prepared for the following screening (**Figure 1**).

**Table 1:** List of the search keywords.

Related to	Sample search keywords in the publication topic
Diabetic retinopathy	“diabetic retinopathy”, “diabetic macular edema”, “exudates”, “intraretinal microvascular abnormality”, “microaneurysm”, “neovascularization”.
Machine learning	“machine learning”, “deep learning”, “supervised learning”, “unsupervised learning”, “adversarial learning”, “classification”, “neural networks”, “predictive model”, “random forest”, “decision trees”, “pattern mining”, “support vector machine”, “multitask learning”, “probabilistic graphical model”, “association rules”.

### Screening strategy

As we searched based on a relatively big set of keywords, some irrelevant documents may also be retrieved. Three authors (A.S., K.J. and L.L.) made the inclusion criteria by reviewing the first 500 documents (primary screening). The practical inclusion criteria: 1. Involves ML technologies. 2. Involves DR, including: (1) Studies focused on DR; (2) Studies focused on the characterized clinical features of DR, for example, microaneurysms and exudates detection (20); (3) Studies focused on multiple diseases and DR was included (21); (4) Studies focused on a topic that is beneficial to the various clinical scenarios of DR, for example, blood-vessel segmentation in the fundus images is beneficial for the following diagnosis of DR (22). After carefully reviewing the 2960 retrieved documents (secondary screening by A.S. and K.J.), 1147 documents were included for the bibliometric analysis (**Figure 1**).

### Bibliometric analysis

Bibliometric analysis was conducted on 1147 documents for obtaining insights into the current trends and topics on ML for DR. In this study, we conducted a trend analysis of publications and citations, publication pattern and collaboration analysis, research domains and targeted sources analysis, as well as the analysis of the keywords.

The analytic tool of the WoSCC database, and Microsoft Excel were used for data mining and representation. The summarized data included publication count and citation count of years/countries/institutions/journals/research domains. Self-citations were included. The Hirsch-index (H-index) was used originally to reflect the academic impact of a researcher, which describes that a researcher has published *h* number of articles, and each of the *h* articles has at least *h* times of citations (23, 24). Currently, the H-index is commonly used for assessing the academic influence of countries/institutions/journals in the bibliometric analysis (25). The growth rate of publications was calculated as follows:


$$Growth\;rate=\left(\sqrt[{t_{2}-t_{1}}]{p_{2}÷p_{1}}-1\right)\times 100$$

where t_1_: First year; t_2_: Last year; p_1_: Publication count of the first year; p_2_: Publication count of the last year.

### Visualized analysis

VOSviewer and Wordcloud were also applied to visualize the collaboration of countries/institutions, co-occurrence of keywords and evolutions of hotspots in the target field. VOSviewer is an analytical tool for constructing and displaying bibliometric maps in an easy-to-interpret manner. To use VOSviewer, we first exported the entire record and cited references of included documents in plain text form, and then the data were imported into VOSviwer (version 1.6.17) (26). By adjusting the options of types and other parameters (type of analysis: co-occurrence; unit of analysis: author keywords; counting method: full counting; the size variation of items, labels and lines between two items were also adjusted for the best presentation), we generated the primary bibliometric maps. Based on the observation of these maps, we generated a text file of the thesaurus to avoid the appearance of synonyms (e.g., “automated detection” & “automatic detection”) in the keyword map. At last, certain meaningless keywords (e.g., “level”) were also deleted to generate the final diagram. To use Wordcloud, we loaded the Wordcloud Python package. The data of title, abstract and keywords were exported and stored as 5 text documents corresponding to 5 periods of time. The thesaurus file was also applied so that synonyms will be regarded as the same word/phrase. After deleting the meaningless characters in the files (e.g., “TI”, the abbreviation before each title), we generated 5 diagrams, representing the 5 studied periods.

## Results

### Trend analysis of publications and citations

**Figure 2:**
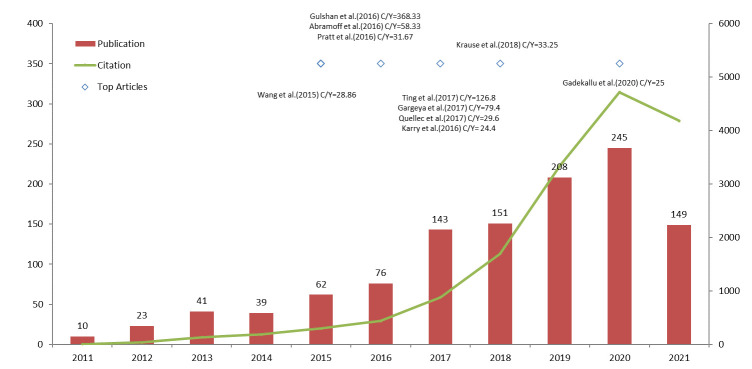
Trend analysis of publications and citations.

**Figure 2** plots the annual trends of publications and citations on machine learning in diabetic retinopathy. We included 1147 articles for the analysis in this study (658 journal articles, 449 proceedings and 40 reviews). From 2017, the annual publication number exceeded 100, and the last 5 years (2017-2021) contributed 78.12% (896/1147) of all articles. The average growth rate from 2011 to 2020 was 42.68%. Polynomial regression analysis was conducted to model the publication and citation trends (2021 was excluded because of incomplete indexing). The estimated models of y1 = 2.6591x2 - 3.2742x + 15.433 and y2 = 102.63x2 - 670.7x + 911.78 indicate changes in the quantities of publications and citations with time, respectively. Both the results of the growth rate and polynomial regression model demonstrate the significant and rapid increase in publications and citations, indicating that machine learning in diabetic retinopathy keeps gaining researchers’ attention and the field is generally at the growth phase. The detailed publication number of different article types and study designs (retrospective/prospective) during 2011-2021 were shown in **Table 2**.

**Table 2:** The yearly publication count.

Year	Article type			Count	Study Design	
	Journals	Proceedings	Reviews		Retrospective	Prospective
2011	3	7	0	10	0	0
2012	12	10	1	23	2	0
2013	19	20	2	41	3	0
2014	22	17	0	39	3	0
2015	26	36	0	62	3	0
2016	26	47	3	76	7	1
2017	59	84	0	143	7	3
2018	73	75	3	151	18	3
2019	111	85	12	208	20	8
2020	181	51	13	245	38	16
2021	126	17	6	149	23	11
Total	658	449	40	1147	124	42

Moreover, we listed the top 10 articles ranked by annual citation count in **Table 3**. Of these 10 articles, all were journals, and 6 articles were published in the last 5 years. The earliest article was by Wang et al. in 2015 (33), introducing a new retinal blood-vessel segmentation method that was beneficial to the screening of DR. The most impactful article was by Gulshan et al. in 2016 (14). They developed a deep learning algorithm to identify referable/non-referable DR and DME, which was a milestone in this field. The algorithm achieved fairly high performance with the area under the receiver operating curve above 0.99 in 2 publicly available datasets (EyePACS and Messidor-2). It is noteworthy that the medical device called IDx-DR mentioned in the pragmatic trial by Abramoff et al. is the first device authorized for marketing by the FDA to automatically detect DR based on fundus images without the need for the interpretation of an additional specialist. The year 2016 and 2017 witnessed 7 of the top 10 articles in this field. The top 10 articles ranked by total citation count were also listed in **Table 3**. Three journals and one review published before 2015 were newly on the list.

**Table 3:** Top articles ranked by annual citations and total citations.

References	Title	Year	C/Y	Source Title
Top 10 articles ranked by annual citations (C/Y)				
Gulshan et al. (14)	Development and Validation of a Deep Learning Algorithm for Detection of Diabetic Retinopathy in Retinal Fundus Photographs	2016	368.3	JAMA
Ting et al. (27)	Development and Validation of a Deep Learning System for Diabetic Retinopathy and Related Eye Diseases Using Retinal Images From Multiethnic Populations With Diabetes	2017	126.8	JAMA
Gargeya et al. (28)	Automated Identification of Diabetic Retinopathy Using Deep Learning	2017	79.4	Ophthalmology
Abramoff et al. (29)	Improved Automated Detection of Diabetic Retinopathy on a Publicly Available Dataset Through Integration of Deep Learning	2016	58.3	IOVS
Krause et al. (30)	Grader Variability and the Importance of Reference Standards for Evaluating Machine Learning Models for Diabetic Retinopathy	2018	33.3	Ophthalmology
Pratt et al. (31)	Convolutional Neural Networks for Diabetic Retinopathy	2016	31.7	Procedia Computer Science
Quellec et al. (32)	Deep image mining for diabetic retinopathy screening	2017	29.6	Medical Image Analysis
Wang et al. (33)	Hierarchical retinal blood vessel segmentation based on feature and ensemble learning	2015	28.9	Neurocomputing
Gadekallu et al. (34)	Early Detection of Diabetic Retinopathy Using PCA-Firefly Based Deep Learning Model	2020	25	Electronics (Switzerland)
Karri et al. (35)	Transfer learning based classification of optical coherence tomography images with diabetic macular edema and dry age-related macular degeneration	2017	24.4	Biomedical Optics Express
References	Title	Year	TC	Source Title
Top 10 articles ranked by total citations (TC)				
Gulshan et al. (14)	Development and Validation of a Deep Learning Algorithm for Detection of Diabetic Retinopathy in Retinal Fundus Photographs	2016	2210	JAMA
Ting et al. (27)	Development and Validation of a Deep Learning System for Diabetic Retinopathy and Related Eye Diseases Using Retinal Images From Multiethnic Populations With Diabetes	2017	634	JAMA
Gargeya et al. (28)	Automated Identification of Diabetic Retinopathy Using Deep Learning	2017	397	Ophthalmology
Abramoff et al. (29)	Improved Automated Detection of Diabetic Retinopathy on a Publicly Available Dataset Through Integration of Deep Learning	2016	350	IOVS
Mookiah et al. (36)	Computer-aided diagnosis of diabetic retinopathy: A review	2013	217	Computers in Biology and Medicine
Wang et al. (33)	Hierarchical retinal blood vessel segmentation based on feature and ensemble learning	2015	202	Neurocomputing
Pratt et al. (31)	Convolutional Neural Networks for Diabetic Retinopathy	2016	190	Procedia Computer Science
Antal et al. (37)	An Ensemble-Based System for Microaneurysm Detection and Diabetic Retinopathy Grading	2012	171	IEEE Transactions on Biomedical Engineering
Srinivasan et al. (38)	Fully automated detection of diabetic macular edema and dry age-related macular degeneration from optical coherence tomography images	2014	168	Biomedical Optics Express
Zhang et al. (39)	Exudate detection in color retinal images for mass screening of diabetic retinopathy	2014	151	Medical Image Analysis

### Publication pattern and collaboration analysis

**Figure 3:**
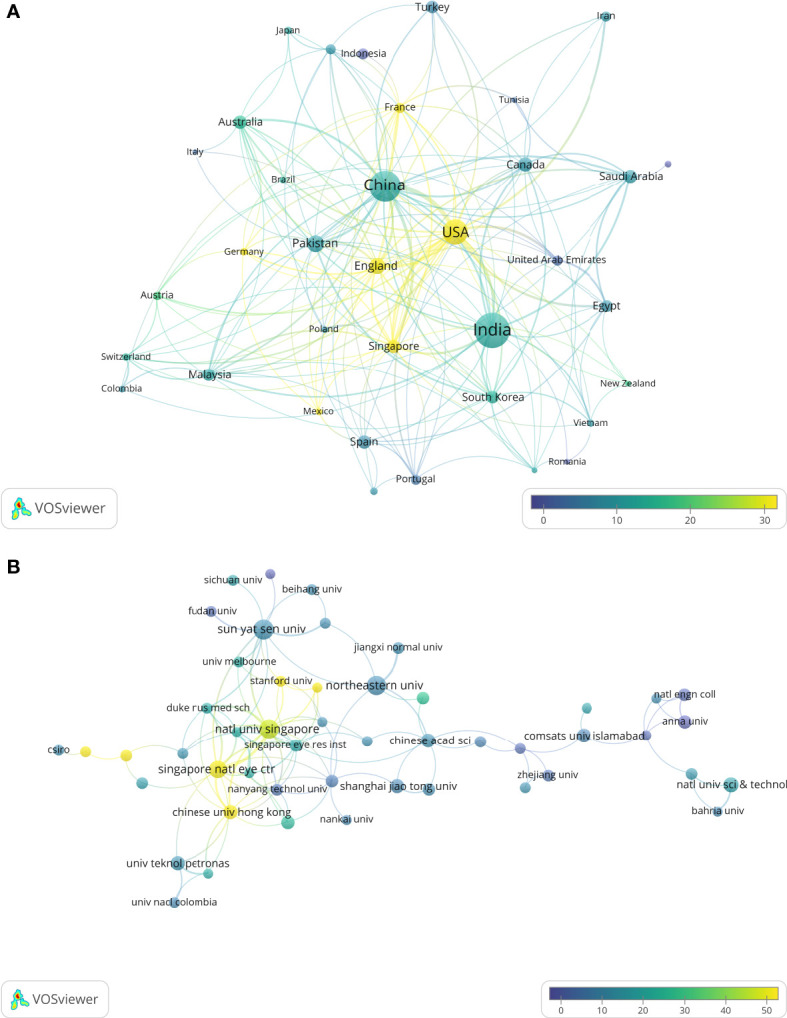
Collaboration maps between countries and institutions.**(A)** Highly contributed countries. **(B)** Highly contributed institutions. Circle size represents the publication count; circle color represents average citations; links represent the collaboration.

Overall, 58 countries contributed to the publications on this topic. The top 10 countries ranked by total publication output accounted for 92.50% (1061/1147) of all included studies and were listed in **Table 4**. India published the most documents (350/1147), accounting for 30.51% of all included studies. China was the second leading country (222/1147, 19.35%), followed by the USA (161/1147, 14.04%). It is worth noting that the USA ranked 1st in terms of citation count while it ranked 3rd in the publication count, and the citation ranks of Singapore, Malaysia, and Australia were also higher than their publication ranks. Institutions with at least 15 documents were also listed in **Table 4** ranked by total publications. National University of Singapore is the most prolific institution (26/1147, 2.27%), followed by National University of Sciences Technology, Pakistan (25/1147, 2.18%) and Singapore National Eye Center (24/1147, 2.09%). However, the publication of reviews cannot directly indicate the active research of a certain institution. Therefore, we also calculate the number of publications except for the reviews. National University of Sciences Technology was the most active institution in research (23/1147, 2.01%), followed by Sun Yat-Sen University (22/1147, 1.92%). There were 3 institutions from Singapore with the highest overall H-index (11, 14, 14). Three Chinese institutions, 2 Indian institutions, 2 American institutions and 2 Pakistan institutions were listed in **Table 4**. **Figure 3** demonstrates the collaboration networks of countries (documents ≥5, 36 countries were included) and institutions (documents ≥5, 65 institutions met the criteria, 16 institutions had no connections to other institutions and were excluded, hence 49 institutions were included).

**Table 4:** Top countries and institutions ranked by publication count.

Country	Documents	%	Citations (Rank)	H-index	Article type		
					Journals	Proceedings	Reviews
India	350	30.57	4183(2)	25	185	159	6
China	222	19.39	2732(3)	26	162	55	5
USA	161	14.06	6393(1)	31	101	55	5
Pakistan	66	5.76	936(7)	17	43	19	4
England	58	5.07	1610(5)	17	40	13	5
Singapore	49	4.28	1909(4)	20	34	9	6
Canada	42	3.67	435(9)	12	22	19	1
Malaysia	40	3.49	1102(6)	18	25	13	2
Australia	37	3.23	663(8)	16	23	12	2
Saudi Arabia	36	3.14	358(10)	12	31	4	1
Institution (Country)	Documents	%	Citations	H-index	Article type		
					Journals	Proceedings	Reviews
National University of Singapore (Singapore)	26	2.27	1075	14	14	7	5
National University of Sciences Technology Pakistan (Pakistan)	25	2.18	599	12	13	10	2
Singapore National Eye Center (Singapore)	24	2.09	1082	14	17	4	3
Sun Yat-Sen University (China)	22	1.92	899	8	18	4	0
Indian Institute of Technology System (IIT System) (India)	20	1.74	218	6	11	9	0
Northeastern University China (China)	20	1.74	209	7	12	8	0
COMSATS University Islamabad (CUI) (Pakistan)	19	1.66	224	9	13	2	4
National Institute of Technology (Nit System) (India)	18	1.57	76	4	9	9	0
Chinese Academy of Sciences (China)	17	1.48	175	5	13	4	0
Ngee Ann Polytech (Singapore)	16	1.4	777	11	14	0	2
Mansoura University (USA)	15	1.31	118	4	9	6	0
University of Louisville (USA)	15	1.31	148	6	8	7	0

### Research domains and targeted sources

**Table 5** shows the 10 most common research domains that the included documents belong to. Computer Science (537/1147, 46.82%), Engineering (527/1147, 45.95%) and Radiology Nuclear Medicine Medical Imaging (128/1147, 11.16%) were the 3 main research domains.

**Table 5:** The most related research domains ranked by publication count.

Research Domain (WoS categories)	Count^a^	%
Computer Science	537	46.82
Engineering	527	45.95
Radiology Nuclear Medicine Medical Imaging	128	11.16
Telecommunications	116	10.11
Medical Informatics	97	8.46
Imaging Science Photographic Technology	93	8.11
Ophthalmology	85	7.41
Mathematical Computational Biology	71	6.19
Optics	70	6.10
Science Technology Other Topics	47	4.10
^a^ Some documents belong to multiple research areas.		

Journals with H-index ≥5 and publications ≥10 were listed in **Table 6**, ranked by publication count. We also referred to the Journal Citation Reports (JCR)(2020) to demonstrate the academic impact of these journals. *IEEE Access* was the journal with the most articles published (36, 3.14%) while *IEEE Transactions on Medical Imaging* was the most impactful among all included journals at the time of analysis. The top 12 journals, which only accounted for 5.31% of 226 journals that have published articles in this field, published 29.23% of all journal articles and reviews (204/689). Seven journals ranked “Q1” in JCR, two journals ranked “Q2” and one journal ranked “Q3”. As for the conference documents, only the *International Conference on Medical Imaging Computer-Aided Diagnosis* published more than 5 documents (7/1147, 0.61%).

**Table 6:** Most productive journals ranked by publication count.

Source Title	Count	Citations	H-index	%	Journal Citation Reports 2020	
					Impact	Rank
IEEE ACCESS	36	399	11	3.14	3.367	Q2
LECTURE NOTES IN COMPUTER SCIENCE	32	196	7	2.79		
COMPUTER METHODS AND PROGRAMS IN BIOMEDICINE	19	652	13	1.66	5.428	Q1
COMPUTERS IN BIOLOGY AND MEDICINE	18	750	14	1.57	4.589	Q1
TRANSLATIONAL VISION SCIENCE TECHNOLOGY	16	110	6	1.40		
JOURNAL OF MEDICAL SYSTEMS	15	411	9	1.31	4.460	Q1
BIOMEDICAL OPTICS EXPRESS	14	599	9	1.22	3.372	Q2
PLOS ONE	13	356	9	1.13	3.240	Q1
IET IMAGE PROCESSING	11	99	5	0.96	2.373	Q3
COMPUTERIZED MEDICAL IMAGING AND GRAPHICS	10	373	9	0.87	4.790	Q1
ARTIFICIAL INTELLIGENCE IN MEDICINE	10	191	8	0.87	5.326	Q1
IEEE TRANSACTIONS ON MEDICAL IMAGING	10	403	8	0.87	10.048	Q1

### Keywords analysis

**Figure 4:**
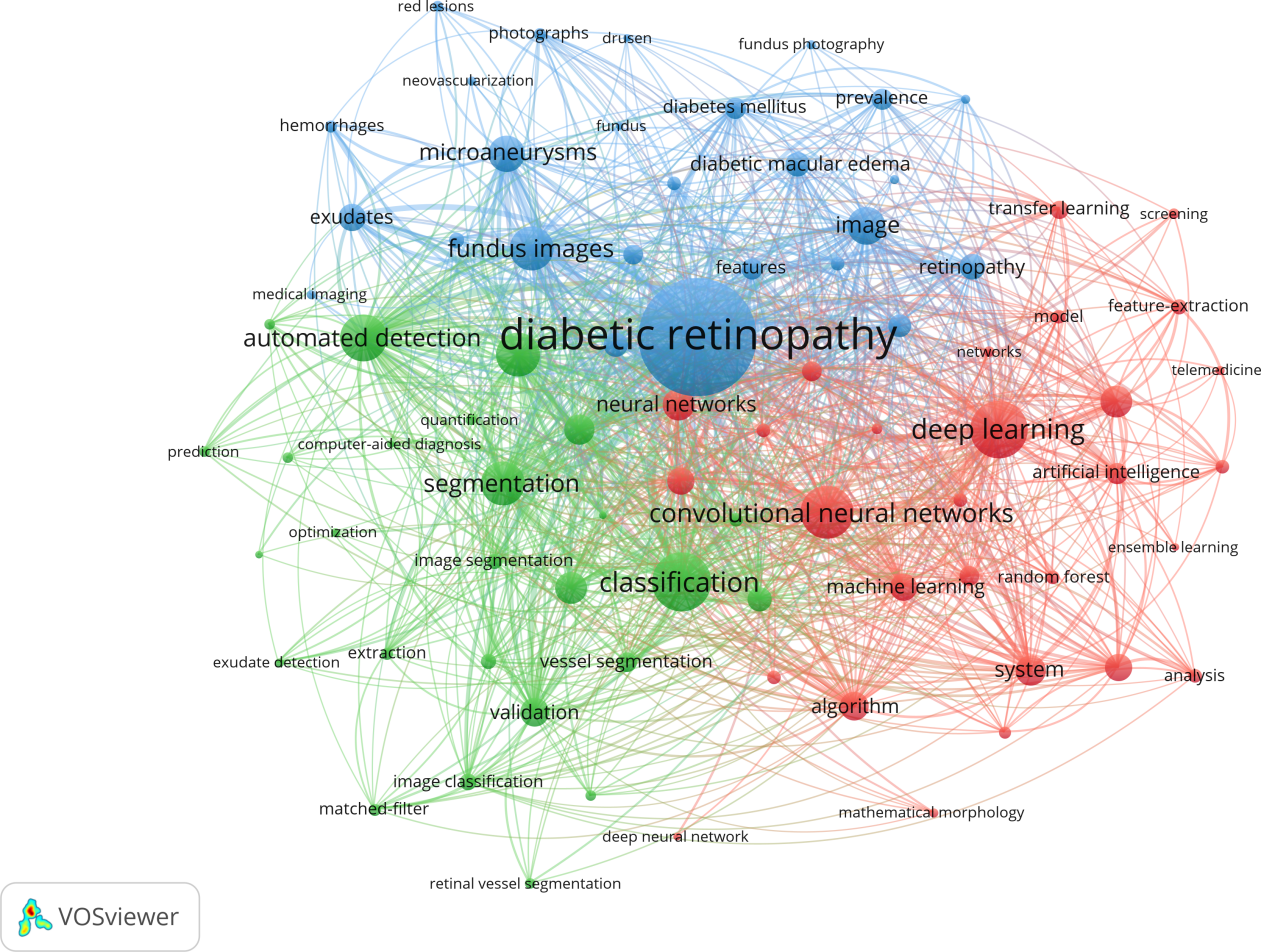
The co-occurrence map of keywords; reveals 3 clusters (in 3 colors): ML techniques; applications of ML techniques; relevant diseases, clinical features and medical data. Circle size represents the frequency of occurrence; links represent the co-occurrence.

To obtain a deeper understanding of research topics and how they are interconnected, we visualized the hotspots of included studies by conducting a keyword co-occurrence analysis using VOSviewer (**Figure 4**). For the total of 2088 automatically identified keywords, 84 keywords occurred at least 10 times, which were shown in **Figure 4**. This map of keywords illustrates the hotspots related to machine learning in diabetic retinopathy. All included keywords were divided into 3 clusters, indicated by red, green and blue colors, representing the ML techniques (e.g., “deep learning”, “convolutional neural networks”, etc.), applications of ML techniques (e.g., “classification”, “segmentation”, etc.) and the DR-related diseases, clinical features and medical data (e.g., “microaneurysms”, “fundus images”, etc.), respectively. From **Figure 4**, we can identify the hot topics represented by strongly linked keywords and the weakly-explored subareas between 2 relatively isolated keywords.

**Figure 5:**
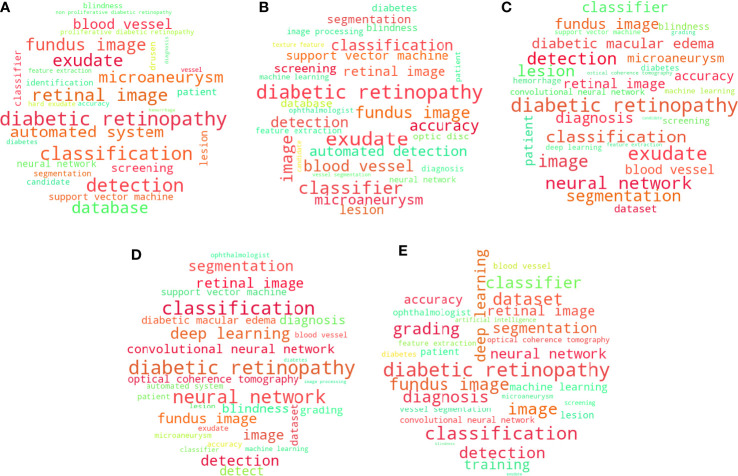
Cloud maps of keywords in 5 periods (the top 30 most frequently-occurred keywords in each map, red keywords are more dominant, green keywords are less dominant): **(A)** during periods 2011-2013; **(B)** during periods 2014-2015; **(C)** during periods 2016-2017; **(D)** during periods 2018-2019; **(E)** during periods 2020-2021.

To understand when these hotspots emerged and how they evolved, we divided the documents into 5 groups by publication time:1.2014-2015; 2.2016-2017; 3.2018-2019; 4.2020-2021; 5.2011-2013 (the only 3-year group, considering that in the first 3 years, the academic output was relatively small when compared with other periods). Five corresponding maps of keywords were conducted by Wordcloud (**Figure 5**). Each map includes the top 30 keywords ranked by the frequency of occurrence. The size of the font represents the frequency (the more frequently-occurred, the bigger scale). “Diabetic retinopathy” was the most dominant keyword for the entire period. Other frequent keywords included “classification”, “fundus image”, “deep learning”, indicating that most studies in this field focused on applying the classification ability of ML techniques into DR based on the medical images. **Figure 5A** displays the top keywords identified during 2011-2013, where the dominant keywords besides “diabetic retinopathy” were “microaneurysm”, “exudate”, and “blood vessel” (ranked 1st to 15^th^, red color), whereas “neural network”, “diagnosis” and “database” were less dominant (ranked 16th to 30^th^, green color). In 2014-2015, “detection”, “segmentation”, and “support vector machine” were more dominant, while “blindness”, “image processing”, and “vessel segmentation” were less dominant (**Figure 5B**). In 2016-2017, “detection”, “neural network” and “diabetic macular edema” were more dominant; “deep learning”, “convolutional neural network”, and “support vector machine” were less dominant (**Figure 5C**). In 2018-2019, “deep learning”, “optical coherence tomography” and “dataset” were more dominant; “exudate”, “microaneurysm” and “grading” were less dominant (**Figure 5D**). In 2020-2021, “deep learning”, “convolutional neural network” and “grading” were more dominant; “lesion”, “blood vessel” and “exudate” were less dominant (**Figure 5E**). The detailed frequency of keywords was listed in **Supplementary Table 1**.

## Discussion

### Trend analysis of publications and citations

From 2011 to 2020, the number of publications grew from 10 to 245 and the overall growth rate reached 42.68%, indicating significant growth in research interests in this field. In addition, the rapid expansion of the annual citations reflected the increasing impact of related publications. On the one hand, this growing trend is due to the breakthroughs in AI technology and its wide application in medicine: in 2012, a well-trained deep convolutional neural network won the ImageNet challenge (40); in 2014, the generative adversarial network was invented (41). As a subarea of ML, DL was gradually applied to various domains of medicine, including radiology, pathology, dermatology, ophthalmology, and so on (42). On the other hand, multiple public ophthalmic datasets were set up around 2010, which accelerated the development of relevant research. For example, Kaggle EyePACS (2015) consists of over 80000 annotated fundus images with DR staging; the Messidor dataset (2008) consists of 1200 fundus images accompanied with medical diagnosis. These public large-scale datasets have created a great opportunity for academic groups worldwide to test and benchmark their models/systems/algorithms. Furthermore, the establishment of recognized DR grading standards (e.g., ICDRSS scale) (7) also promoted the comparison of diagnostic ability between different models or between man and machine. In general, there is still a distance between the current ML in DR and the clinical practice as most studies are in silico and aim to optimize algorithms and propose new techniques based on recognized public datasets or local private datasets. The prospective studies in this field mainly focus on the real-world viability test, clinical validation of algorithms/software and human-machine comparison (43–45). However, as machine learning becomes mature in this area, the number and proportion of real-world-oriented studies are increasing.

**Table 3** shows that the most impactful articles were published after 2015. After Gulshan et al. published the most impactful in 2016 and received widespread attention from ophthalmic researchers, many DL-based studies have sprung up, which is also consistent with the publication trend and the development of technologies and databases. Some common points of impactful articles were found out: 1. Published by influential journals (e.g. JAMA - IF:56.27; Ophthalmology, IOVS – the top journals of ophthalmology); 2. New techniques (e.g. deep learning in 2016, 2017); 3. Excellent results (e.g., great performance of algorithms with an area under the receiver operating curve > 0.99); 4. Involved in multiple tasks (e.g. automatic grading of DR severity or detection of multiple diseases including DR). These articles led the developing trend in this field and many articles were based on these achievements.

### Publication pattern and collaboration analysis

Researchers all over the world have contributed to the field of ML in DR. The publication pattern reveals that India and China have been the most productive countries. The two densely populated developing countries contributed to nearly half of the relevant publications, which is uncommon in other bibliometric studies on the topic of AI technologies in medicine (17, 25), as developed countries such as the USA or England are usually the main force. In addition, there are 5 developing countries in the top 10 countries ranked by publication count, all with considerable academic output. However, in terms of the H-index and citations of different countries, developed countries performed relatively better compared to developing countries. This can be explained by differences in social medical resources and technologies between countries. With the global epidemic of diabetes, the prevalence of DR is also rising predominantly, especially in densely populated countries like India and China (46, 47). There is a clear but unmet need to comprehensively screen DR in the diabetic population in the rural area of these developing countries due to the disproportionally low ophthalmic population (13). Developing countries are urgently calling for a cost-effective way to manage DR. Therefore, the automatic system based on ML is widely explored by academic groups from developing countries. As for developed countries, institutions and researchers benefit from technological breakthroughs and the mature ophthalmic system. Researchers are more likely to publish impactful articles. The National University of Singapore is the most productive institution and most publications also belong to Singapore National Eye Center. The two institutions tend to publish articles that push forward the clinical application of ML techniques in DR, including the clinical validation of DL systems based on the Singapore National Diabetic Retinopathy Screening Program or other multiethnic DR screening data and reviews that discussed the current status of AI techniques in the real-word DR screening (48, 49). By analyzing the top institutions (documents ≥ 20), we found that most studies from National University of Sciences Technology Pakistan, Indian Institute of Technology System and Northeastern University China are ML technique-oriented. Most studies from two Singaporean institutions are medicine-oriented. Researchers from Sun Yat-Sen University published both technique-based studies and clinical validation studies as they collaborated a lot with hospitals and computer science laboratories. From the perspective of citations, those medicine-oriented and pragmatic studies are more popular than technique-oriented studies.

The collaboration analysis also revealed that productive countries/institutions have more options for international collaborations. In addition, the nodes in the middle of **Figure 3A** tend to appear yellow, indicating that countries/institutions with more external collaborations have a greater chance of publishing impactful articles (i.e., high average citations).

### Research domains and targeted sources

As included documents are mainly related to computer techniques and imaging systems, journals that specialized in these domains were productive in this field. On the one hand, the advancement of computer science and engineering accelerated the pace of applying AI technologies in medicine. On the other hand, imaging techniques are commonly used in ophthalmology and produce lots of valuable data on DR patients, which is useful for developing ML algorithms. The impact factor (mostly around five) and the JCR rank of the twelve “core journals” indicate the overall impact and quality of relevant publications. Only *Computers in Biology and Medicine* and *Biomedical Optics Express* have published impactful articles, as shown in **Table 3**. Impactful journals such as *JAMA* and *Ophthalmology* are not shown due to the publication count.

### Keywords analysis

The frequently occurred keywords in the literature always indicate the research hotspots. The co-occurrence of several keywords represents the widely discussed topic containing several basic components. By dividing the relevant literature by time, the emergence and evolution of keywords can be visualized on the word clouds. Keywords analysis reveals the mainstream topics in the field, the research focuses on different periods and the subareas that are currently popular or remained to be explored.

Overall, the application of machine learning techniques in diabetic retinopathy is extensive and diverse, while most documents aim to diagnose DR automatically. “Diabetic retinopathy” is the most dominant keyword for the whole period, along with other frequent keywords such as “classification”, “segmentation” and “fundus images”. Thus, fundus images are the most commonly used data for research. Classification and segmentation are the tasks for ML or the processing steps for the data. Some keywords relating to DR lesions (e.g., “microaneurysms”, “exudates”) are also dominant in **Figure 5**, as many documents focus on detecting characterized lesions of DR to mimic the diagnostic process of ophthalmologists. A tiny microaneurysm can be the key to distinguishing between diseases and normality, thereby the automatic detection of these lesions makes the diagnosis of ML algorithms reasonable (20). Some keywords of ML techniques were prominent in the keywords co-occurrence analysis (e.g., “deep learning”, “support vector machine”, “convolutional neural network”), representing the popular tool applied in DR. By linking up the keywords, the mainstream concepts are immediately visible, for example, the diagnosis of “diabetic retinopathy” based on the “automated detection” of “exudates” in “retinal images” by “deep learning”.

However, both techniques and clinical focuses change over time. From 2011 to 2021, the evolution of topics mainly focused on computer methods, clinical tasks and data modalities. First, “deep learning” and “convolutional neural network” appeared in 2016-2017 for the first time and subsequently became larger in the word cloud, indicating that deep learning and related techniques gained increased research attention, which was consistent with the publication time of the paper by Gulshan et al. and the overall development of DL techniques. The traditional technique “support vector machine” became less popular in this field due to the remarkable performance of DL in feature extraction and representation. Second, the keywords of DR features (e.g., exudate, microaneurysm) became less frequent, indicating that simple lesion-detection algorithms were gradually dismissed. Many comprehensive DR grading systems and multi-disease diagnosis systems have sprung up recently as the keyword “grading” gradually become frequent (21). Third, due to the limited information offered by digital fundus images, the data from new imaging techniques such as optical coherence tomography, gradually emerged in this field (**Figures 5C–E**). Other imaging techniques like fundus fluorescein angiography were also considered but not shown in the word cloud, which needs to be further studied (50). Moreover, the ML algorithms usually focused on the simple data modality while doctors would refer to different types of examination data and complaints of patients. As the keyword “dataset” and “database” has become much more dominant from 2011 to 2021, the integration of multi-modal data from different sources might be the future direction for automated diagnosis. In addition, we found that although the keyword “patient” was less prominent from 2011 to 2021, the frequency rank kept rising. From a clinical point of view, patients are always the main components of all relevant studies. With the ML techniques in DR getting matured, more researchers designed studies that better reflect the real-world effectiveness of AI systems. These studies not only included the existing datasets but also test their algorithm/software in broader patient groups. To utilize AI as tools in real clinical settings, the algorithms in this field are constantly optimized in both the techniques (from “support vector machine” to “deep learning”) and the capacity of dealing with more complex conditions which mimic the clinical settings (e.g., grading DR based on multi-modal data).

This study is the first bibliometric analysis of ML in DR and aims to provide a holistic view of the relevant research. The results discussed in this study are objective, quantifiable and macroscopical, which would be suitable for any researchers interested in this field to get familiar with the basic knowledge structure (e.g., the mainstream topic, the outstanding achievements, the emerging trends, global publication pattern, and relevant research domains, etc.) and can help them find potential collaborators and develop relevant studies. Moreover, the change in publication trends and keywords from 2011 to 2021 indicated the potential directions of further studies in this field, including the incorporation of optimized ML techniques, multi-modal data, real-world-oriented study design, etc.

## Limitations

This study has some limitations. First, we only used reference data from a single database (WoSCC) and the results of the bibliometric analysis may not be as robust as studies that collect data from multiple databases due to some unpredictable bias when we search for documents in WoSCC. However, WoSCC is a well-indexed database that represents one of the largest multidisciplinary collections of indexed published literature. Moreover, the list of keywords may not be comprehensive enough to retrieve all related documents even if we referred to the relevant literature and books. Second, although some meaningless keywords were deleted in the figure conducted by VOSviewer and Wordcloud, not all keywords are informative enough in the figure, such as “system”. These general keywords occur frequently but do not refer to any deeper subfields, therefore, these keywords cannot be analyzed. Finally, like other bibliometric analyses, this study didn’t focus on the content of every single article; the uniqueness and novelty of most articles were ignored and only top articles were analyzed. Third, the emerging novel topics discussed in this study may have stagnations to practice as the co-occurrence maps of keywords are based on frequency. A breakthrough was reflected on these maps only when it gradually became recognized in the research community and also it takes time for researchers to cite these articles (to be listed as top articles in the bibliometric analysis).

## Conclusions

In this study, we provided a comprehensive overview of all retrieved articles on ML in DR following a bibliometric approach for the first time. It’s a growing research area and has been studied by researchers from multiple countries and institutes. As new topics have emerged and evolved since 2011, studies in this field are becoming more diverse and extensive. Real-world-oriented studies with multi-modal data and optimized ML techniques are the further directions as clinical application is the ultimate goal in this field. Further studies can focus on larger research fields (e.g., AI techniques in ophthalmology) and the integration of data from multiple databases.

## Data availability statement

The original contributions presented in the study are included in the article/**Supplementary Material**. Further inquiries can be directed to the corresponding authors.

## Author contributions

Design of the work: AS, KJ, WZ and JY. Collection and screening of the data for the work: AS, KJ and LL. Analysis of the data: YL. Drafting the work: AS. Revising of the manuscript: KJ, LL, WZ and JY. All authors contributed to the article and approved the submitted version.
