# Supplementary material for: Overview of global publications on machine learning in diabetic retinopathy from 2011 to 2021: Bibliometric analysis
Source: Front Endocrinol (Lausanne). 2022 Dec 15;13:1032144. doi: 10.3389/fendo.2022.1032144 (PMC9797582; doi:10.3389/fendo.2022.1032144)
Supplement: Supplementary file 1 [file Table_1.docx]

| **Supplemental Table 1.** The frequency of top 30 keywords in five periods. | | | | | | | | | |
| --- | --- | --- | --- | --- | --- | --- | --- | --- | --- |
| **2011-2013** | **Frequency** | **2014-2015** | **Frequency** | **2016-2017** | **Frequency** | **2018-2019** | **Frequency** | **2020-2021** | **Frequency** |
| **More frequent keywords** | | | | | | | | | |
| diabetic retinopathy | 227 | diabetic retinopathy | 306 | diabetic retinopathy | 627 | diabetic retinopathy | 1036 | diabetic retinopathy | 1150 |
| classification | 119 | image | 195 | classification | 329 | neural network | 413 | deep learning | 521 |
| microaneurysm | 117 | classification | 165 | detection | 272 | fundus image | 401 | image | 460 |
| fundus image | 116 | exudate | 157 | image | 254 | classification | 396 | fundus image | 446 |
| detection | 109 | fundus image | 126 | fundus image | 253 | image | 325 | classification | 434 |
| retinal image | 85 | detection | 121 | retinal image | 179 | deep learning | 305 | neural network | 429 |
| exudate | 70 | retinal image | 108 | microaneurysm | 172 | detection | 303 | detection | 318 |
| blood vessel | 53 | classifier | 71 | blood vessel | 162 | retinal image | 276 | dataset | 285 |
| automated system | 50 | blood vessel | 70 | exudate | 154 | segmentation | 267 | segmentation | 281 |
| screening | 49 | segmentation | 60 | neural network | 150 | convolutional neural network | 239 | convolutional neural network | 249 |
| support vector machine | 48 | accuracy | 58 | segmentation | 144 | optical coherence tomography | 227 | diagnosis | 236 |
| vessel | 45 | support vector machine | 54 | diabetic macular edema | 123 | dataset | 208 | grading | 228 |
| segmentation | 44 | microaneurysm | 54 | accuracy | 107 | diabetic macular edema | 208 | retinal image | 225 |
| lesion | 39 | screening | 53 | dataset | 94 | blood vessel | 204 | optical coherence tomography | 223 |
| classifier | 36 | lesion | 51 | diagnosis | 92 | exudate | 199 | accuracy | 222 |
| **Less frequent keywords** | | |  |  |  |  |  |  |  |
| diabetes | 33 | automated detection | 46 | classifier | 89 | accuracy | 195 | patient | 181 |
| proliferative diabetic retinopathy | 32 | database | 43 | lesion | 86 | support vector machine | 195 | blood vessel | 176 |
| blindness | 31 | neural network | 43 | patient | 78 | microaneurysm | 190 | machine learning | 176 |
| diagnosis | 29 | diagnosis | 43 | convolutional neural network | 74 | diagnosis | 185 | microaneurysm | 166 |
| neural network | 29 | blindness | 41 | blindness | 71 | patient | 167 | lesion | 165 |
| accuracy | 28 | diabetes | 41 | deep learning | 71 | classifier | 134 | diabetes | 151 |
| hard exudate | 27 | image processing | 35 | diabetes | 69 | lesion | 131 | classifier | 121 |
| identification | 26 | patient | 33 | screening | 66 | blindness | 114 | training | 113 |
| database | 25 | optic disc | 32 | support vector machine | 63 | grading | 110 | ophthalmologist | 112 |
| patient | 25 | machine learning | 32 | candidate | 62 | automated system | 106 | screening | 110 |
| non proliferative diabetic retinopathy | 23 | texture feature | 31 | machine learning | 60 | diabetes | 101 | vessel segmentation | 107 |
| candidate | 21 | feature extraction | 28 | optical coherence tomography | 60 | image processing | 87 | feature extraction | 106 |
| feature extraction | 20 | ophthalmologist | 26 | feature extraction | 57 | detect | 86 | artificial intelligence | 105 |
| hemorrhage | 19 | candidate | 26 | hemorrhage | 51 | ophthalmologist | 81 | blindness | 103 |
| drusen | 19 | vessel segmentation | 25 | grading | 48 | machine learning | 79 | exudate | 102 |
